# *Hypericum perforatum* and Its Potential Antiplatelet Effect

**DOI:** 10.3390/healthcare10091774

**Published:** 2022-09-15

**Authors:** Maria-do-Céu Monteiro, Alberto C. P. Dias, Daniela Costa, António Almeida-Dias, Maria Begoña Criado

**Affiliations:** 1 TOXRUN-CESPU Toxicology Research Unit, University Institute of Health Sciences, CESPU, CRL, 4585-116 Gandra, Portugal; 2 CITAB-UM-Centre for the Research and Technology of Agro-Environmental and Biological Sciences, Department of Biology, Campus de Gualtar, University of Minho, 4710-057 Braga, Portugal; 3 IA &HEALTH-CESPU Research Unit in Artificial Intelligence and Health, CESPU, CRL, 4585-116 Gandra, Portugal

**Keywords:** *Hypericum perforatum*, platelet activation, flow cytometry, traditional Chinese medicine

## Abstract

Background: *Hypericum perforatum* (HP) is currently one of the most consumed medicinal plants in the world. In traditional Chinese medicine, the herb hypericum (*Guan Ye Lian Qiao*) belongs to the group of plants that clarify heat. It is also used to treat various types of infection and inflammation. In contrast to the extensive literature on the antidepressant effects of HP, little is known about its action on platelets. The main objective of this work was to investigate the possible relevance of HP to platelet function. Methods: We characterized the profile of platelet activation in the presence of HP extracts through an evaluation of molecular markers by flow cytometry: mobilization of intracellular Ca++ and expression of platelet receptors such as activated GPIIbIIIa and P-selectin (CD62). Results: The results indicated a possible inhibitory effect of HP on the platelet activation response, which could be explained by the effect on intracellular calcium mobilization and the expression of activated GPIIbIIIa receptors. Despite of the limitations of an in vitro study, our results provide evidence of the possible mechanisms of action of HP. Conclusions: Further studies are needed to elucidate the effect of HP on hemostasis, but it may be recognized as a substance with antiplatelet properties.

## 1. Introduction

Hypericum or St. John’s wort has a long tradition of use as a medicinal plant. It is included in the pharmacopeia of many regions [1]. In Europe, this species is one of the oldest medicinal plants, being documented as early as ancient Greece. The therapeutic properties of HP were first described by Hippocrates. St John’s wort species have been traditionally used for their astringent, antipyretic, diuretic, analgesic and, mainly, antidepressant effects [1,2,3]. In traditional Chinese medicine (TCM), hypericum, as a heat-clarifying *materia medica*, has traditionally been prescribed, alone or in combination with other plants, not only for treating depression, but also to eliminate toxins, in hemoptysis and hematemesis, and to disperse external moisture-heat and relieve muscle pain [4,5,6]. However, most of these traditional uses still do not have scientific support, and more studies are needed to confirm the therapeutic bioactivities attributed to HP.

Several biologically active substances in HP have been described. To date, seven classes of active compounds have been identified: naphthodianthrones, phloroglucinol derivatives, proanthocyanidins, essential oils, xanthines and flavonoids. Up to about 20% of extractable substances are considered to be biologically active, with naphthodianthrones, including hypericin and pseudohypericin, being the most studied group [3,7].

Currently, St. John’s wort is used primarily in the treatment of anxiety, depression, and sleep disorders [8]. The market of products derived from St. John’s wort has grown to international proportions, with a value of approximately $250–300 million. The strongest market is in Germany [9]. However, other therapeutic applications have also been described, such as antibacterial, antiviral, anti-inflammatory, antioxidant and neuroprotective, among others [3]. From these applications, the most studied in relation to the mechanism of action is the antidepressant properties. Several studies have shown that hypericin has a strong affinity for sigma receptors that regulate dopamine levels. Hyperforin also seems to give HP its antibacterial power, as this compound has the ability to inhibit the growth of some species of microorganisms including gram-negative bacteria and methicillin- and penicillin-resistant Staphylococcus aureus [7,10,11]. The flavonoids present in HP extract confer antioxidant and neuroprotective properties by decreasing the oxidation of the mitochondrial lipid membrane and maintaining the transmembrane electrical potential of this organelle. On the other hand, its anti-inflammatory effect on tissues may contribute to the induction of nitric oxide synthesis [12].

Regarding the possible effect of St. John’s wort on platelet function, little is known.

Platelets primarily function to participate in hemostasis and thrombosis. Following vascular injury, platelets become activated, resulting in adhesion to the exposed extracellular matrix, the formation of a platelet plug, and finally, the formation and consolidation of a thrombus. Activated platelets show altered expression of various surface glycoproteins (GP). Activated platelets expose GPIIb-IIIa that undergoes conformational changes by activation before binding to fibrinogen, which is a key phenomenon for thrombus growth. Additionally, in this activation phase, P-selectin, an adhesion molecule stored in the α granules of platelets, is translocated to the surface.

Various disease conditions such as diabetes, atherosclerosis, hypertension and heart disease show a common risk characteristic, i.e., high thrombus formation due to increased platelet activation. Also, activated platelets could be directly involved in the unstable plaque through the production and release of pro-inflammatory molecules, including a variety of cytokines such as TGF-β, IL-1β and sCD40L, among others [13]. Thus, antiplatelet therapy has been used for a long time in an effort to prevent, as well as to treat, thrombotic diseases.

An increased risk of bleeding has been attributed to the use of certain herbs, including St. John’s wort, and some in vitro experiments have identified St. John’s wort extracts as platelet inhibitors [14]. Additionally, it is well established that platelet activation by agonists such as serotonin result in a cascade of platelet degranulation and aggregation mediated by the release of intracellular calcium stores [15]. Given that hyperforin is one of the principal components of HP and is known to inhibit serotonin uptake, it can be speculated that HP can alter platelet activation. In the same way, another component of HP with antioxidant properties may also alter its platelet function. As alterations in platelet activation have been associated with pathologic conditions, leading to either thromboembolic or hemorrhagic disorders, in the framework of healthcare, it is relevant to assess the effect of HP on hemostasis disorders [16]. As such, it is necessary to carry out in vitro studies related to its mechanisms of action, prioritizing studies related to traditional uses. In this sense, the biological effects of *Hypericum perforatum* on platelets need to be elucidated in order to contribute to the evaluation of its therapeutic potential. Thus, the main objective of this work is to investigate the possible functional relevance of *Hypericum perforatum* in platelet activation.

## 2. Materials and Methods

### 2.1. Obtaining HP Extracts

The high parts of a HP plant were used to obtain the extracts. All plants were collected from the Braga region (Portugal). The plants were dried for 5 to 7 days. With the dry material, extraction was performed with an ethanol:water solution (80:20) according to the method previously described by Silva et al. [17]. After obtaining TAE dry extracts in a vacuum, the TAE dry extracts were obtained and resuspended in DMSO. Chromatograms were obtained at 260, 350 and 590 nm. The composition of the total alcoholic extract (TAE) obtained was characterized by HPLC-DAD, revealing the total hyperforin, chlorogenic acid, total flavonoid and total hypericin contents.

### 2.2. Selection of Donors

Whole blood samples were collected from normal individuals (n = 5), aged between 18 and 45 years, with no personal or family pathology of recognized hemostasis and with no antiplatelet medication use in the two weeks prior to blood collection. All donors presented normal platelet aggregation responses to agonists (ADP and collagen). Other exclusion criteria were smokers, hypertensive patients and patients with type I or II diabetes.

### 2.3. Blood Collection and Treatment of Samples with Hypericum perforatum Extract

To avoid artifacts in platelet activation, whole blood was collected without a tourniquet from the antecubital vein, and phlebotomy was performed with a 19-gauge needle. The first 2.5 mL was discarded, and 9 mL was collected directly into a tube containing 1 mL of sodium citrate (129 mM).

Whole blood samples were incubated with different concentrations of total ethanolic extract of HP (0–0.500 mg/mL) for 15 min at room temperature.

### 2.4. Kinetics of Intracellular Calcium Mobilization

The kinetics of intracellular calcium mobilization, as well as the determination of the expression of activated GPIIbIIIa receptors and P-selectin (CD62P), were analyzed by flow cytometry. Flow cytometry enables studies of several different aspects of platelet function in response to a variety of platelet agonists and allowed us to perform a real-time functional analysis of platelets using only a small volume of whole blood.

Intracellular calcium mobilization was measured by the method described by Monteiro et al. [18]. Briefly, whole blood previously treated with HP was diluted (1:10) in Thyrode buffer (TB) and incubated with Fluo-4 (5 µM) (15 min, room temperature) and then labeled with saturating concentrations of the monoclonal antibody CD41-PE to identify the platelet population. Tetrapeptide GPRP (2.5 mM) was added to inhibit fibrin polymerization. After 15 min of incubation at room temperature in the dark, TB (1 mL) was added and 500 µL samples were used to determine α-thrombin-induced intracellular calcium mobilization. The flow cytometer (EPICS XL, Coulter), with laser emission at 488 nm, was set to measure forward light scatter (FLS), side light scatter (90LS) and fluorescences, Fluo-4 (FL1) and PE (FL3), using logarithmic amplification mode. In the double-labeled samples, Fluo-4 fluorescence determinations were analyzed in the window of CD41(+) events. After determination of baseline Fluo 4 fluorescence (log FL1), thrombin (0.05 U/mL) was added and changes in log FL1 vs. TIME were recorded.

### 2.5. Determination of the Expression of Activated GPIIbIIIa Receptors and P-Selectin (CD62P)

Surface expression of activated platelet receptors GPIIbIIIa and P-selectin was determined by flow cytometry in whole blood by modified methods previously described [19] using monoclonal antibodies PAC-1 (Becton Dickinson), which bind to the activated conformation of the GPIIbIIa complex and anti-CD62P (IMMUNOTECH) directed against P-selectin. Briefly, HP-treated whole blood samples were diluted (1:10 in TB) and platelets stimulated with Thrombin 0.05 U/mL after inhibition of fibrin polymerization with GPRP. The samples were then doubly labeled with saturating concentrations of PAC 1-FITC/CD61-PE or CD62P-FITC/CDCD41-PE monoclonal antibodies. After this incubation, 1 mL of TB was added, and the samples were analyzed using a flow cytometer. The expression of activated GPIIbIIIa and CD62 receptors was determined in the CD61(+) or CD41(+) events, respectively. In parallel, 25 µL of unstimulated blood was incubated with the labeled antibodies as described above to assess basal activation.

### 2.6. Ethics

All blood samples were collected with the informed consent of the volunteers. During the study, the recommendations contained in the Declaration of Helsinki (with the amendments of Tokyo 1975; Venice 1983; Hong Kong 1989; Somerset West 1966 and Edinburgh 2000 and the World Health Organization) were respected, with regard to experimentation involving human beings. Ethics committee approval was not required, as no human samples were identified and this was not an interventional study [20].

## 3. Results

Table 1 shows the different compounds found in the dry extract of the HP plant. As shown, the HPLC spectra of the HP extracts revealed the presence of a large number of flavonoids.

**Figure 1:**
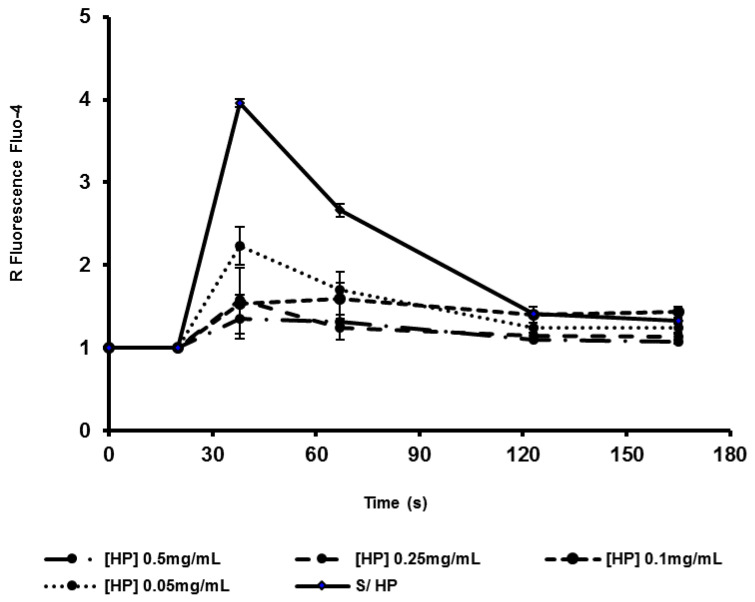
Effect of HP on the kinetics of free cytosolic Ca ++ after stimulation with thrombin (0.05 U/mL). of whole blood platelets. Data are presented as mean +/− SEM for three different experiments with the same donor. Fluorescence ratios = mean FL1 intensity in agonist-stimulated platelets/mean FL1 intensity in unstimulated platelets.

**Figure 2:**
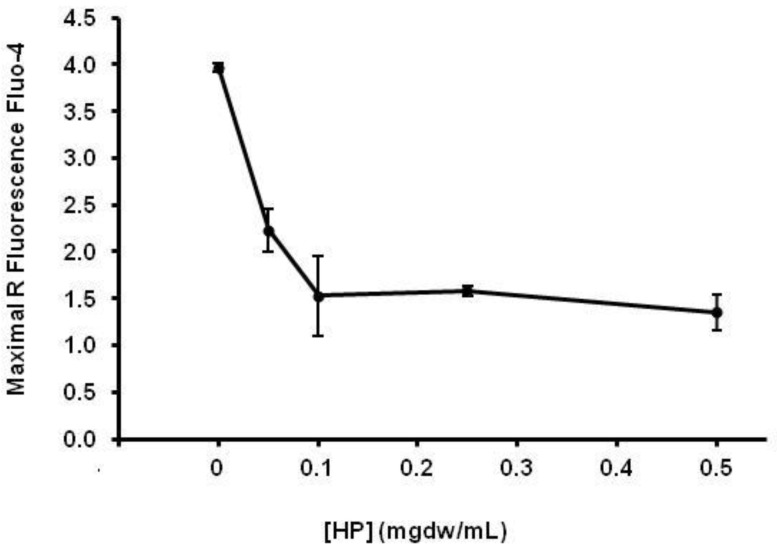
Effect of HP on maximal free cytosolic Ca++ response after stimulation of whole blood platelets with thrombin 0.05 U/mL). Fluorescence ratios = mean FL1 intensity in agonist-stimulated platelets/mean FL1 intensity in unstimulated platelets. Data are presented as mean +/− SEM for three different experiments with the same donor.

**Figure 3:**
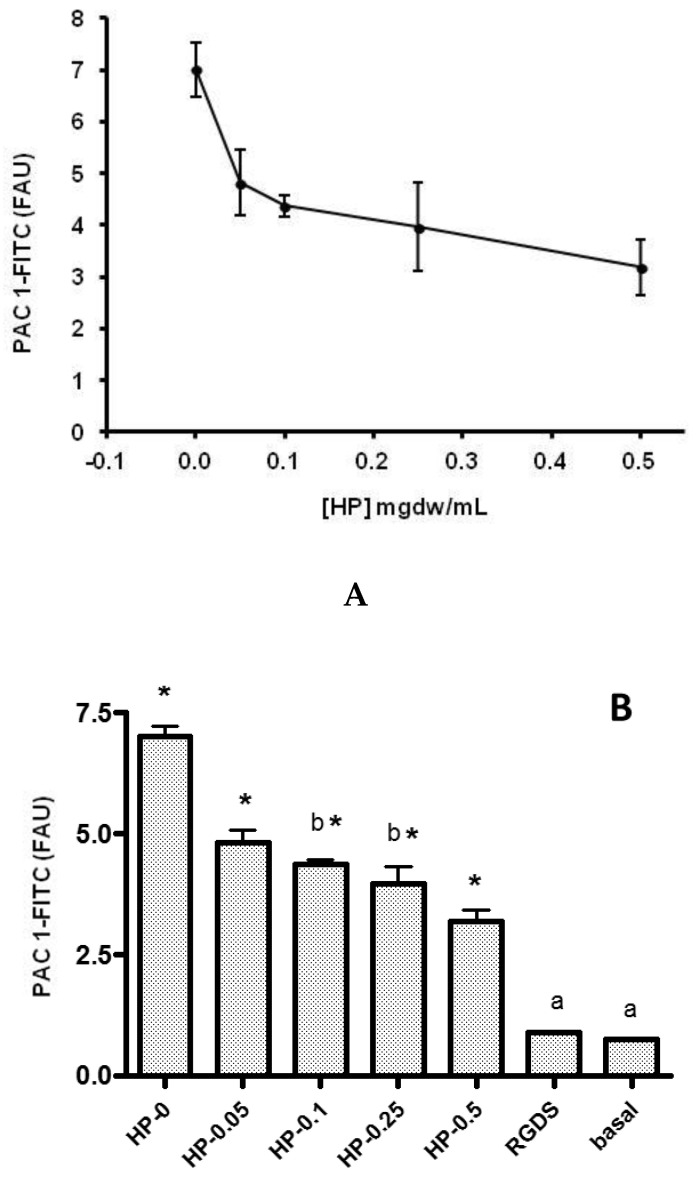
Effect of HP on thrombin-induced expression of activated GPIIbIIIa (0.05 U/mL) assessed by fluorescence intensity. Data are presented as mean+/− SEM for three different experiments with the same donor. FAU: PAC1-FITC-fluorescence units in total platelet population (CD6+ events) (**A**). (**B**) Global HP effect and statistical comparison. All values obtained with HP are significantly different from those obtained without treatment (*), except those marked with the same letter (a and b).

**Figure 4:**
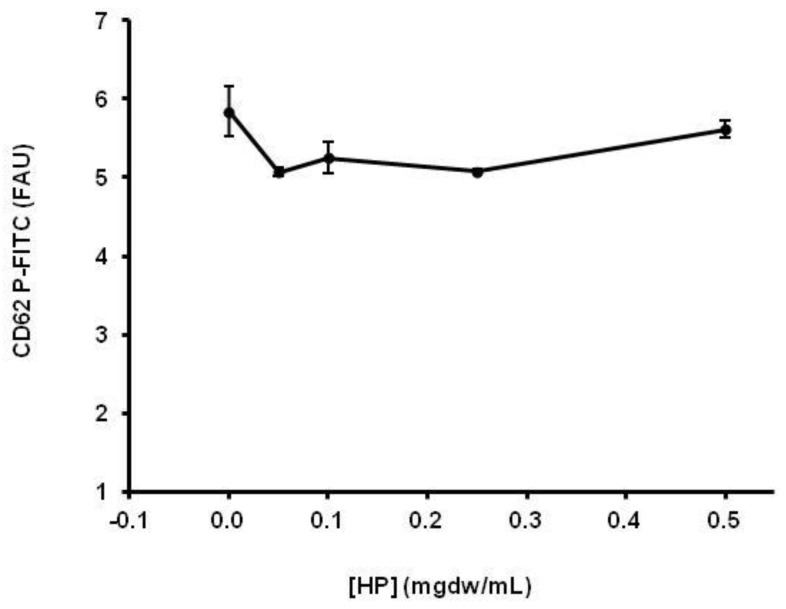
Effect of HP on thrombin-induced CD62P expression (0.05 U/mL), assessed by mean fluorescence intensity. Data are presented as mean +/− SEM for three different experiments with the same donor. FAU: CD62P-FITC-fluorescence units in the total platelet population (CD41+ events). The values were not statistically different.

An ethanolic extract of HP was used for the in vitro analysis of molecular markers of platelet activation by flow cytometry. The main results are shown in Figure 1, Figure 2, Figure 3 and Figure 4. The platelet surface expression of activated GPIIbIIIa glycoprotein (fibrinogen receptor) and P-selectin were studied using the PAC-1 and anti- CD62 antibodies, respectively. For all HP concentrations used, we observed a clear inhibitory response: the ethanolic HP extract inhibited the expression of activated GPIIbIIa (inhibition > 50%) and the expression of P-selectin in whole blood platelets. Regarding the mobilization of intracellular Ca++, an inhibitory effect of the HP extract was also observed. This effect was observed for all extract concentrations, and the observed inhibition was dependent on the HP dose.

## 4. Discussion

According to the WHO [21], herbal medicine is one of the therapies with the greatest and growing public demand, with HP being one of the most consumed medicinal plants in the world due to its biological activities. Notably, HP has been widely used to treat depression [8]. In 2008, a review by Cochrane concluded that the results using plant extracts of HP were superior to placebo in patients with major depression, and were equally effective as standard antidepressants with fewer side effects [22,23]. Also, antioxidant, vasoprotective, anticarcinogenic, and antiviral properties of HP have been described [24,25].

In TCM, HP extracts have also been used for centuries to treat depressive and behavioral disorders. However, *Guan Ye Lian Qiao* (Hypericum herb), historically considered an acidic, bitter, astringent and “neutral” plant and a “heat-clarifying” *materia medica*, has also been used for other purposes, such as eliminating toxins, cooling heat blood pressure and against infection and inflammation. For example, *H. attenuatum* is used to treat heart disease in northeast China [4]. In TCM, plants that clarify the heat of the blood calm its circulation, but at the same time, they must prevent its stagnation, so they must not be too cold-natured [26]. The herb hypericum, as a “neutral” plant, has a stabilizing (astringent), tonifying, harmonizing (antispasmodic) action, balancing *Qi* and blood, and therefore, is suitable in these conditions.

The present work investigated the possible functional relevance of *Hypericum perforatum* in platelet function, related to its uses in TCM. To achieve this goal, we evaluated the ex vivo effect of HP on platelet reactivity using flow cytometry methods to analyze the biochemical markers of platelet activation. Flow cytometry is described as a powerful tool to perform platelet functional analysis that allows the investigation of basal and agonist-induced platelet functional responses [27] In our study, the antiplatelet effects of HP were explored by testing their activity on different early activation-dependent events in human platelets: intracellular calcium mobilization, the presence of activated GPIIbIIIa receptors and of P-selectin in platelet surface.

We found that HP displays in vitro antiplatelet activities, and that this inhibitory effect is directly proportional to the concentrations used. Intracellular Ca++ mobilization responses and activated GPIIbIIIa (fibrinogen receptors) expression were strongly reduced after HP treatment, resulting in an inhibition greater than 50%. Few scientific studies have been carried out on the antiplatelet or anti-inflammatory actions of HP, but some have shown that, like other herbal medicines such as garlic, ginger, ginseng, and willow bark, HP can reduce platelet aggregation by reducing available intracellular calcium [28]. Our results also provided evidence that the mechanism of action of HP may be related with intracellular calcium mobilization.

Platelet activation plays an important role in cardiovascular disease, the leading cause of mortality and morbidity in developed countries. In addition, platelet activation is related to platelet aggregation responses in hemostasis. In this sense, platelets are known to share similar biochemical processes with neurons. The processes of serotonin capture, storage and release in platelets are kinetically and pharmacologically similar to those in the central nervous system. Thus, serotonin transporters in the human brain and platelets are identical. In this sense, hyperforin was one of the first substances observed to inhibit serotonin uptake by increasing Na. Since serotonin is an agonist of platelet aggregation, it could be speculated that the inhibition of serotonin uptake by *Hypericum perforatum* may also be an important mechanism in decreasing the signal of platelet recruitment during the aggregation process. According to this, some studies showed an inhibition of HP in platelet aggregation [29].

The main limitations of our study are related the use of ex vivo analyses and our study of only the biochemical markers of platelet activation. In future works, it will be necessary to extend the study to a larger sample of donors, to deepen in the mechanisms of inhibition of platelet activation, including the study of the expression of Ca-related proteins, and to study the effect of HP in platelet aggregation responses. Most in vivo studies of HP have been dedicated to the use St. John’s wort in depression. With respect to HP and platelets, in vivo studies are very scarce [14], and the majority of them have sought to determine the possible interactions between herbal medicines, including St. John’s wort, and prescribed drugs [30]. Taking into account the promising results obtained in our study, further randomized, double blind, placebo-controlled crossover trials on the effect of HP on platelet function and coagulation profile in healthy volunteers are necessary.

An important aspect that should also be studied is related to drug interactions, since it is known that these can occur due to the fact that St. John’s wort induces either P-glycoprotein or some cytochrome P450 isoforms, such as CYP3A4 [31]. P-glycoprotein acts as a carrier across intracellular and extracellular membranes. St. John’s Wort causes the expression of this protein to be positively regulated, resulting in a decrease or increase in the effectiveness of other drugs taken at the same time. The CYP3A4 isoform is responsible for metabolizing more than 50% of commercially available drugs [32]. Most of the studies carried out in this regard showed that St. John’s wort induces the expression of several CYP450 isoforms, causing pharmacokinetic changes and, therefore, its therapeutic effect, namely, in the case of anticoagulant therapy, which can increase the risk of bleeding in patients under these therapies [31]. It is important to mention that Chinese herbal medicine is mainly based on the use of formulas with medicinal plants that act in synergy to produce a therapeutic effect and to reduce adverse effects. Unlike the western approach, which mainly acts on one therapeutic target, Chinese herbal medicine focuses on several targets and has several mechanisms of action simultaneously. TCM has been developed over thousands of years to increase the efficiency and decrease the toxicity of herbs [33].

## 5. Conclusions

The effects of HP on hemostasis need to be elucidated in order to evaluate its therapeutic potential, but our results seem to indicate that HP may be recognized as a substance with antiplatelet properties and, therefore, could be considered useful in the prevention of cardiovascular diseases.

## Figures and Tables

**Table 1:** Compounds found in the dry extract of the HP plant.

KERRYPNX	Compound	µg/mg Dry Extract
Total hyperforins (41, 191)	Hyperforin	33.661
	Adhyperforin	7.530
Total Flavonoids (53, 514)	Chlorogenic acid	4.108
	Rutin	7.049
	Hyperoside	19.631
	Isoquercetin	6.429
	Ramnosyl-quercetin	7.484
	Hyperoxide-acetil	2.219
	Acetyl-Rutin	0.535
	Quercetin	6.128
	Biapigenin	2.136
	Quercetin-3-O-b-D-glucuronic acid	1.003
Total Hypericins (1, 414)	Protopseudohypericin	0.175
	Pseudohypericin	0.499
	Protohypericin	0.171
	Hypericin	0.570

## Data Availability

Not applicable.
